# Titanium-Based Hip Stems with Drug Delivery Functionality through Additive Manufacturing

**DOI:** 10.1155/2015/134093

**Published:** 2015-10-04

**Authors:** Martin B. Bezuidenhout, Dimitar M. Dimitrov, Anton D. van Staden, Gert A. Oosthuizen, Leon M. T. Dicks

**Affiliations:** ^1^Rapid Product Development Laboratory, Department of Industrial Engineering, University of Stellenbosch, Stellenbosch 7600, South Africa; ^2^Department of Microbiology, University of Stellenbosch, Stellenbosch 7600, South Africa

## Abstract

Postoperative infections are a major concern in patients that receive implants. These infections generally occur in areas with poor blood flow and pathogens do not always respond to antibiotic treatment. With the latest developments in nanotechnology, the incorporation of antibiotics into prosthetic implants may soon become a standard procedure. The success will, however, depend on the ability to control the release of antibiotics at concentrations high enough to prevent the development of antibiotic-resistant strains. Through additive manufacturing, antibiotics can be incorporated into cementless femoral stems to produce prosthetic devices with antimicrobial properties. With the emerging increase in resistance to antibiotics, the incorporation of antimicrobial compounds other than antibiotics, preferably drugs with a broader spectrum of antimicrobial activity, will have to be explored. This review highlights the microorganisms associated with total hip arthroplasty (THA), discusses the advantages and disadvantages of the latest materials used in hip implants, compares different antimicrobial agents that could be incorporated, and addresses novel ideas for future research.

## 1. Introduction

**Figure 1:**
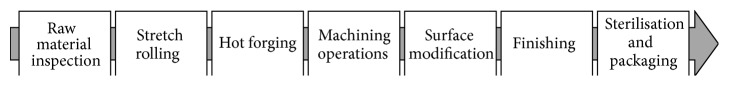
Simplified process chain for titanium alloy cementless femoral stems (adapted from [19]).

The increased use of cementless femoral components in THA [1], combined with the increased occurrence of THA [2], advocates a significant and growing market for these devices. Most cementless femoral stems are produced from wrought titanium alloys [3], by using conventional manufacturing processes that include rolling, forging, machining, surface modification, finishing, cleaning, and sterilisation (Figure 1). Metal additive manufacturing (AM) is not used in any of the current commercial cementless hip stem manufacturing processes.

**Figure 2:**
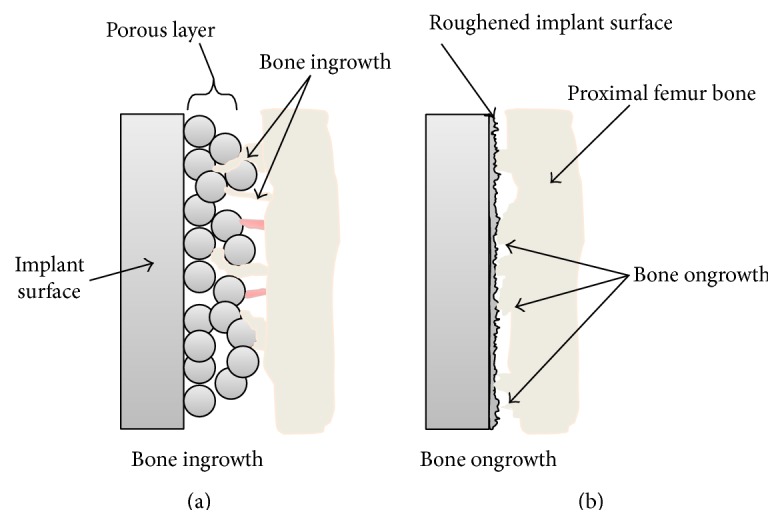
Schematic representation showing the difference between bone ingrowth (a) and bone ongrowth (b).

Proximal surfaces of titanium alloy femoral stems are often coated with plasma spraying to provide a porous surface for bone ingrowth (osseointegration, Figure 2(a)). Although bone ongrowth (Figure 2(b)) also facilitates fixation on a roughened, nonporous surface, it provides less tensile support than bone ingrowth [4].

Clinically approved first-generation wrought titanium alloys have crystal structures consisting of a combination of *α*-phase (hexagonal close packed) and *β*-phase (body centred and cubic) orientations [4]. Second-generation titanium alloy consists mostly of a *β*-phase microstructure with lower elastic moduli, compared to first-generation titanium and cobalt alloys or stainless steel and reduces stress shielding, a phenomenon that occurs due to a mismatch between the elastic modulus of bone and implant material [5]. Of all alloys, titanium is the most used one due to its excellent biocompatibility, resistance to corrosion, high strength, and ductility [4, 6].

Concerns regarding the release of aluminium ions from titanium alloys such as Ti-6Al-4V have been raised. Aluminium has been correlated to the onset of diseases such as Alzheimer's and cytotoxicity has been reported from excessive concentrations of vanadium [7, 8]. These concerns have been mitigated by developing diamond-like carbon (DLC) film that prevents the release of elemental ions from alloys [9].

A serious concern regarding THA devices is the increasing trend in revisions due to infection [3, 10]. According to Kurtz et al. [10], the number of THA infections in the United States of America alone may exceed 16000 by 2020, with an estimated treatment cost of $527.9 million. With a current infection rate of approximately 1% for primary THA, failure to reduce the risk of infection will only lead to increased infection rates during revision surgeries. Furthermore, the burden regarding treatment costs associated with infection will continue to increase. Considering the above, combined with the morbidity and psychological strain put on the patient, it is clear that infection of THA is still a serious issue inhibiting the success of the procedure and the quality of life of many patients.

Selective laser melting (SLM) and electron beam melting (EBM) are powder bed fusion AM processes, during which three-dimensional (3D) parts are formed in a layer-by-layer fashion. This enables the design and manufacturing of a range of products with unique features that were previously difficult or near impossible to assemble. The latest developments in AM triggered a renewed interest in the use of AM technology, especially in the design and production of medical implants with enhanced performance and novel functionalities [11, 12]. The novelty, we believe, lies in the ability to administer multiple dosages of an antimicrobial drug* in situ*, from the implant [13, 14]. By administering the drug directly to the site of infection, postoperative surgery and removal and cleaning of the prostheses may be avoided. If refined, this technology could replace many of the current once-off drug release devices that are either coated with antimicrobial compounds or modified with the incorporation of acrylic drug-loaded bone cements.

Implants produced with AM are used in specific cases to reduce stiffness and patient-specific geometries [15–18]. A cementless hip stem with enhancements such as a built-in drug delivery system requires the design of a new process chain. In this paper the focus falls on the application of AM in the design of cementless hip stems with novel drug delivery properties.

## 2. Infection

Infections acquired from implants are difficult to treat. Antibiotics administered, whether orally or intravenously, do not always reach the implant due to restricted blood flow [21]. The surface of implants, on the other hand, is rapidly covered with proteins and glycoproteins produced by the host [22]. This results in a “conditioned surface” that supports adhesion of bacteria [23]. Once adhered, the bacteria secrete polysaccharides and form biofilms to protect themselves from antibiotics. Destruction of a biofilm is extremely difficult, as shown in the treatment of* Pseudomonas aeruginosa* and* Staphylococcus aureus* biofilms [24, 25]. In both cases the level of antibiotics required for treatment is much higher than the minimum inhibitory concentration (MIC), which is not a practical solution. In severe cases, often seen with prosthetic joints, the only treatment options available include debridement [26], one- or two-stage arthroplasty [27], resection arthroplasty [28], removal of the implant [29], or amputation of the limb [30]. Recent reports highlight the increased risk and occurrence of mortality rates associated with deep chronic infection [30, 31]. Pathogens isolated from prosthetic joint infections (PJIs) after THA and total knee arthroplasty (TKA) are listed in Table 1.

Treatment of infected femoral stems remains an area of debate [32]. The choice between procedures depends on the type of infection, time laps after surgery, damage to tissue and bone, condition of the implant, and miscellaneous factors which are situation specific [27]. One option to prevent the formation of bacterial biofilms is to use antibiotic-loaded bone cement (ALBC, Table 2). Although the method is well established and is an accepted practice, concerns about the development of antibiotic-resistant bacteria, due to the elution of antibiotics at levels below MIC, have been raised [33]. This has prompted investigation into incorporation of alternative antimicrobial compounds into bone cement. van Staden et al. [34] incorporated nisin, a lantibiotic produced by* Lactococcus lactis* subsp.* lactis*, into bone cement and succeeded to control the growth of* S. aureus in vivo* in a mouse model. Campoccia et al. [14] showed that implants coated with silver nanoparticles killed most bacteria within the first few days after surgery and the implant retained its antimicrobial properties for 30 days, with no significant decline in activity.

The latest development is cementless fixation of implants and the elution of antimicrobial compounds from within femoral stems [13]. However, conventional manufacturing processes do not allow for intricate geometries and features of custom-designed hip implants [35]. In a recent study titanium alloy cubes with a novel drug delivery design were constructed from Ti6Al4V ELI (Extra Low Interstitial) powder with LaserCUSING (an AM process) with vancomycin incorporated in channels of the cubes. Controlled release of vancomycin could be achieved with approximately 50% of the vancomycin released within the first 17 h [13]. The authors managed to sustain the delivery of vancomycin for as long as 100 h by reinjecting the channels that were sealed with hydrophilic polyethersulfone membranes. This study proved that refillable implants may be a novel way to control postoperative infections.

**Figure 3:**
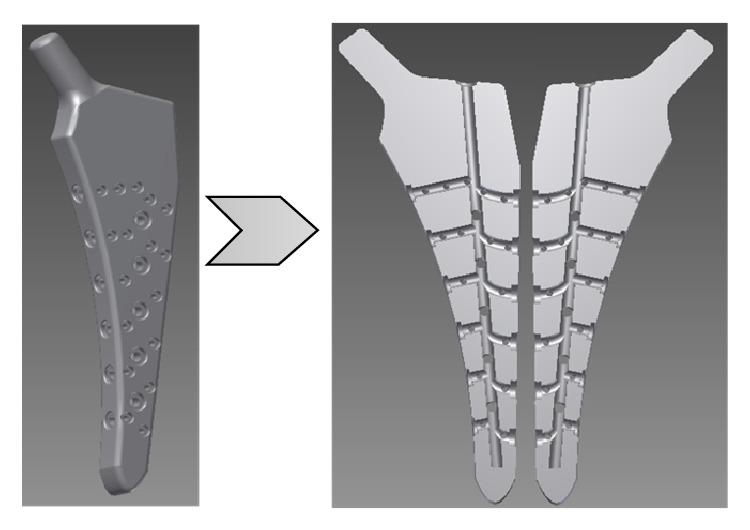
A total hip replacement femoral stem concept with internal channels (adapted from [20]).

Femoral stems used in total hip replacement (THR) therapy are usually manufactured from wrought material by subtractive processes. Mueller et al. [20] used selective laser melting (SLM) to produce a prototype femoral stem similar to that shown in Figure 3. Although EBM (electron beam melting) acetabular cups have been approved [36], heavy load bearing prostheses, such as femoral hip stems manufactured by AM technologies, have not been approved by the United States Food and Drug Administration (FDA) [37] and more extensive clinical trials will have to be performed [38]. The eventual realisation of cementless femoral stems with functional enhancements, such as integrated drug delivery features, could also be beneficial in the treatment of infection. This could mobilise a patient during the interim period of a two-stage exchange or could be used as a permanent implant in a one-stage revision.

## 3. Treatment

Postoperative infection is classified as either an “early” infection that occurs within 2 months after surgery or a “delayed” infection that occurs between 3 and 24 months after surgery [39]. In pursuit of best practices in infection management, Zimmerli and Ochsner [39] developed a decision making algorithm based on time elapsed after surgery, infection type, state of the implant and surrounding tissue, and comorbidity factors. Regardless of the procedure used, success cannot be guaranteed. Infection rates for revision surgeries are typically higher than those recorded for primary arthroplasty. Persistent infections may result in amputation or even death [29, 30].

### 3.1. Early Debridement with Retention

The option of treating a prosthetic joint infection with debridement and retention of the implant is subdued to very strict criteria, for example, stability of the implant, stage of infection, overall health of the patient, and the patient's tolerance to aggressive antibiotic therapy, whether it is administered intravenously or orally [41]. Despite all precautions taken into consideration, infections caused by* S. aureus* remain a problem in THA and TKA [42].

### 3.2. One-Stage and Two-Stage Exchange Arthroplasty

In one- and two-stage exchange arthroplasty, antibiotics are administered for a minimum of two weeks before surgery. One-stage exchange arthroplasty is only performed if pathogens isolated from the infected area were positively identified and if the patient's bone material is still healthy [43]. Two-stage exchange arthroplasty, on the other hand, is performed on patients with abscesses and sinus tracts [39, 44]. In both procedures the infected soft tissue is debrided and the implant, plus accompanying bone cement if present, removed. In one-stage exchange arthroplasty the bone is “keyed” to form a rough surface before the addition of ALBC. The tissue surrounding the infection site is treated with selected antibiotics based on the identified pathogens, the implant is fixed into place, and the wound is closed [45]. If the infection is not eradicated, a two-stage revision is performed, in which case the implant is removed, infectious bone and tissue are debrided, and a temporary spacer of antibiotic-loaded bone cement is implanted to control the infection [39]. If the implant is fixed too solidly, it may be necessary to bivalve the femur to remove the femoral stem [43]. The patient is kept on antibiotics for 3 weeks to 7 months and has limited mobility during this period [40]. The second stage starts with removal of all the antibiotic-loaded beads or bone cement spacer material and testing for the persistence of antibiotic-resistant bacteria [43], in which case the interim period of antibiotic treatment is extended. The new implant, usually composed of cemented components and an ALBC prophylactic, is then inserted [43]. Once the new implant is fixed in place, the wound is closed and the patient is monitored extensively [39]. Buchholtz et al. [45] reported a 77% success rate with one-stage revision. Success rates as high as 86 to 100% were reported when patients were thoroughly screened and pretreated with the correct antibiotics [39]. Two-stage exchange arthroplasty has a higher success rate (>90% success) compared to one-stage exchange arthroplasty [43, 44].

### 3.3. Resection Arthroplasty

Resection arthroplasty is also referred to as modified Girdlestone arthroplasty [53]. The procedure is usually performed on patients with a high surgical risk and not fit to be exposed to one- or two-stage revision arthroplasty [54]. Indications for secondary resection arthroplasty are numerous and include infection with bacteria resistant to several antibiotics, poor condition of surrounding soft tissue, inadequate bone stock, and overall poor health of the patient.

The entire THR prosthesis (femoral and acetabular sections) plus bone cement (in the case of cemented THA) is removed, and the joint space and surrounding tissue are debrided and drained of any abscess and purulence [54]. Resection of the femur is performed at the intertrochanteric line and acetabular osteophytes are removed [53]. The wound is then drained and closed. Ossification normally occurs between the femur and the acetabulum [55]. The disadvantage of this procedure is that the joint is stiff, leaving the patient with considerable disability, and it is therefore only performed as a last resort.

### 3.4. Prophylactic Strategies

The first 6 h postimplantation is considered the most critical when newly implanted material is most vulnerable to infection [60]. It is thus not surprising that prophylactic strategies to control bacterial colonisation on THR femoral stems receive so much attention [60, 61]. Despite many concerns raised about the emergence of antibiotic-resistant bacteria [32, 39] and the use of ALBC in primary cemented hip replacements, antibiotics remain the most used prophylactic treatment in primary and revision arthroplasties [60]. This is also the standard procedure used in the fixation of femoral stems reintroduced in second-stage treatments [43, 60].

Initially, antibiotics were mixed into the poly(methyl methacrylate) (PMMA) matrix at the discretion of the surgeon. This often led to inadequate concentrations of antibiotics added, unpredictable elution characteristics, and adverse effects on the mechanical properties of bone cement [62]. Furthermore, mixing of antibiotics into PMMA did not guarantee even distribution. The range of FDA-approved ALBC cements currently available is listed in Table 2.

**Figure 4:**
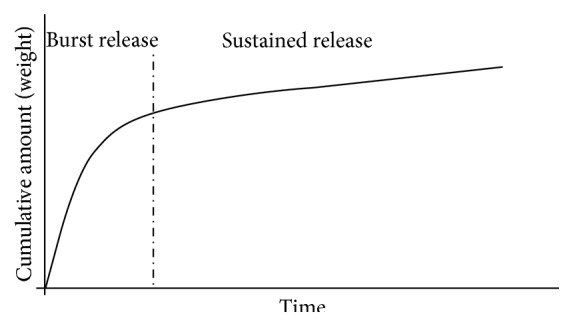
Cumulative release of gentamicin from PMMA (adapted from [56]).

Gentamicin is usually the preferred antibiotic, as it is active against Gram-negative and Gram-positive bacteria [63], remains active when used in combination with other antibiotics, such as vancomycin [33], and is stable at temperatures generated during the exothermic polymerisation of PMMA bone cement [56, 64]. Release of gentamycin from PMMA occurs in two stages; an initial burst release, typically within the first 24 h [62], followed by a steady sustained release for an extensive period [65]. In some reports the transition from burst to sustained release is described as an additional stage [62]. A generic curve showing the cumulative release of gentamicin over time is shown in Figure 4. In an ideal situation, the cumulative concentration of gentamicin released should reach high levels over a short period, followed by almost no release [66].

Lewis [62] studied the release of drugs from implants and related them to different drug delivery mathematical models. Some models do not account for the hydrophobic properties of PMMA and are considered inaccurate [62]. Despite modified versions of drug release models, studies performed with the same CMW1 gentamicin bone cement produced more than one best fit result [66].

A serious problem with the sustained subinhibitory levels of gentamicin release from PMMA is the provocation of antibacterial resistance [60]. In a study by Neut et al. [67] during which gentamicin-loaded PMMA beads were cultured after retrieval from patients, 68% of the 28 identified bacterial strains exhibited resistance towards gentamicin. This further emphasizes the current need for the investigation into more efficacious drug delivery strategies and the thorough integration of the involved processing technologies and disciplines to gain a better understanding towards the eventual development of such combination devices.

## 4. Additive Manufacturing (AM) as Enabler Technology

Several methods have been proposed to prevent bacteria from colonising cementless femoral hip stems [68]. However, most of these methods focused on processing the external surface of the implant. Only a few studies reported on the release of antimicrobial compounds from metallic implants [68]. Linezolid, a synthetic antibiotic, imbedded into mesoporous silica and then incorporated into pores of a 316L stainless steel pin, prevented the colonization of* S. aureus* ATCC 29213 on the surface of the implant [68]. This opened the possibility of incorporating prophylactic antimicrobial compounds and osteoinductive supplements into preformed channels of an implant.

Most of the current literature, however, inevitably results in once-off release strategies without the possibility to tailor drug release after implantation. Furthermore, devices seem to be investigated by adding functionality through postprocessing operations rather than developing integrated devices with multiple drug delivery capabilities [69]. Such products would fall under the FDA regulatory classification of combination devices, which have their own regulatory requirements before clinical acceptance [70].

This creates an area for the development of novel process chains investigating AM processes as enabler technologies. However, in developing these process chains it is important to identify the different roles of stakeholders, especially as these process chains would encompass interdisciplinary communication [71]. Involved parties need to understand the fundamental perspectives across disciplines to collaboratively achieve an efficacious design. This implies moving from a vertical (or line) based perspective, where each party focuses solely on their own expertise, to a more lateral based perspective, where each party understands the fundamental topics from all the other involved disciplines to correctly translate and incorporate them into their own subpart of the design.

Modern AM technologies are based on the paradigm developed in the late 1980s with liquid based stereolithography (SLA), which is considered the cornerstone of rapid prototyping (RP) [72]. Rapid prototyping was initially used to create nonfunctional parts. However, as new technologies emerged, RP evolved into the manufacture of functional parts. These layer-by-layer processes are collectively coined AM [73]. Within these technologies, two powder bed fusion processes, electron beam melting (EBM) and selective laser melting (SLM), are highlighted. In contrary to subtractive processes, in which an object is fabricated by removing of a large volume of starting material, the AM technologies, as the name suggests, are characterised by adding material, thus allowing for the manufacturing of intricate geometries and efficient use of material [58, 73].

According to Cronskär et al. [74], the production cost of highly customised femoral hip stems can be reduced by 35% if AM (electron beam melting, EBM) is used with sufficiently large batch sizes, as a collection of parts with different geometries can be built simultaneously. In another study, Dehoff et al. [75] calculated a 50% cost reduction in the manufacturing of a thin-walled aerospace bracket using AM with appropriate processing conditions. From studies such as these, it is clear that AM technology poses an attractive alternative for manufacturing of new generation drug/device combination hip implants. Metal AM processes such as EBM and SLM can therefore play an important role as enabler technology within the process chains of next generation implants.

**Figure 5:**
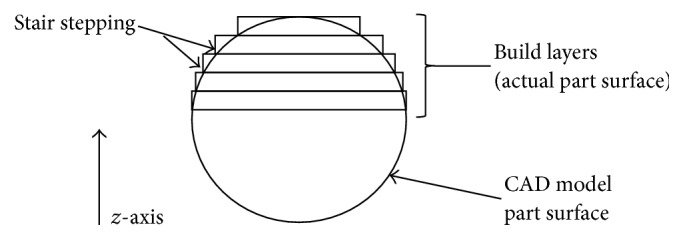
Schematic representation of the stair stepping effect obtained when slicing for finite layer approximation from the original CAD geometry (adapted from [57]).

In general, the AM process consists of (i) generation of a Standard Triangulation Language (STL) file, (ii) file verification and repair, (iii) creation of a build file, (iv) construction of the implant, and (v) cleaning and finishing. Digital manufacturing starts with CAD modelling and export of the design in STL format. Once an STL file is generated, it needs to be verified and defects need to be removed [57]. Creation of the build file requires a number of steps and concepts that have to be taken into consideration. The first step is orientation of the implant. Due to the layer-by-layer nature of AM, all parts will inherently have “stair stepping” (Figure 5), except if all features are completely vertical or horizontal [76]. This effect can be minimised by decreasing the thickness of the layers, which in turn will increase building time. The layering or “slicing” of the implant is specified in the respective software package used during preprocessing of the build file.

Implants are built from the bottom up, one layer at a time. Once the implant is finished, it is removed from the machine, support structures are removed, and the implant is cleaned. Some implants require postprocessing such as wax or bronze infiltration to strengthen the structure. Refining processes are, for example, sand-blasting, grinding, or polishing, and various heat treatments.

### 4.1. Electron Beam Melting

**Figure 6:**
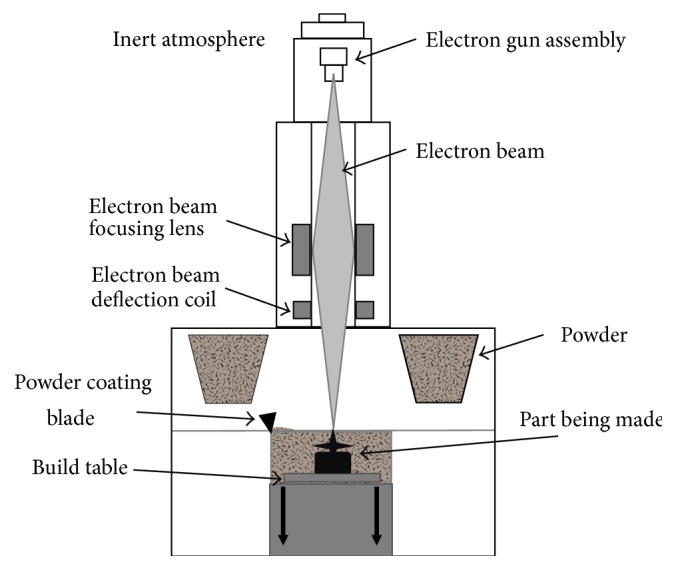
Schematic representation of the EBM process (adapted from [58]).

With EBM, fully functional and nearly fully (>99%) dense metallic parts can be created without the need for additional binder materials [77, 78]. An electron beam in a vacuum is used to melt the metal powders layer-by-layer [58]. A simplified schematic of the EBM process is presented in Figure 6.

The electron beam is supplied with a tungsten filament electron gun, which emits electrons when heated in excess of 2500°C under vacuum [77]. The nominal operating voltage for the electron gun is 60 kV. The electron beam is positioned and controlled by deflection coils to scan and melt the powder on a preheated table to form layers ranging from 0.05 to 0.2 mm [58, 73]. Once a layer is completely scanned, the build table is lowered by the layer thickness and a fresh supply of powder is deposited from the powder depots. A powder coating blade then moves across the build table to ensure an even spread of powder. The layer in question is again scanned by the electron beam and the process is repeated until all layers are formed. Postprocessing operations include machining such as drilling or milling and heat treatment, that is, annealing [79] and hot isostatic pressing (HIP) [80].

### 4.2. Selective Laser Melting

**Figure 7:**
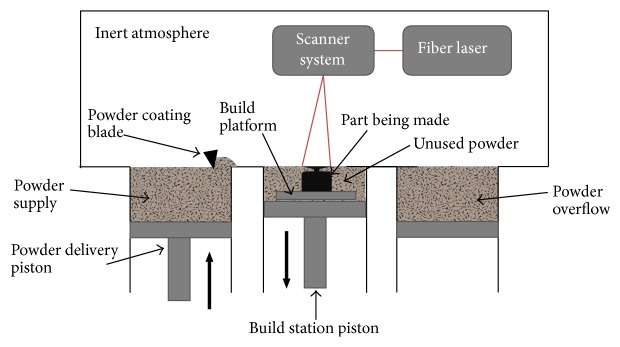
Schematic representation of the SLM process (adapted from [59]).

Selective laser melting (SLM) has been developed more recently and involves the production of complex three-dimensional, near net shape, metallic parts in a layer-by-layer manner. Thermal energy, produced by a focused fiber laser beam, selectively scans, melts, and fuses metallic powder particles on a powder bed, creating near full density (>99%) parts [81, 82]. Part densities as high as 99.81 ± 0.1% have been reported using SLM-processed Ti-6Al-4V ELI [47]. A simplified schematic presentation of the SLM process is shown in Figure 7.

The SLM process, as the name implies, uses energy from a laser beam instead of an electron beam to fuse the powder particles by heating it beyond melting temperatures. A layer of powder is evenly spread across the building plate followed by the scanning of the two-dimensional geometry in question. The build platform then is lowered by the preset layer thickness (typically 30 to 70 *μ*m) [59] before a fresh layer of powder is deposited and evenly spread by the coating blade. Powder not used is recycled [48]. The build chamber is flushed with nitrogen or argon gas to avoid oxidation [76]. Postprocessing includes the removal of support structures, usually by mechanical means such as machining or light chiseling. Further treatment is often necessary, that is, by machining, to reduce roughness or a number of heat treatments procedures aimed at relieving residual stresses, tailoring the microstructure, and reducing porosity [48, 83]. Parts produced by SLM have a layer of partially sintered powder particles around their surface geometries. Markwardt et al. [84] found that osseointegration of human osteoblasts is promoted by these surfaces. The compliance of these surfaces, however, has not yet been tested according to international specifications for medical implants.

## 5. Ti-6Al-4V ELI Powder

A significant percentage of cementless stems are made from Ti-6Al-4V ELI powder. Grade 23 Ti-6Al-4V ELI is widely used in the manufacturing of medical implants and devices.

### 5.1. Surface Texture

As-built parts produced with SLM generally have a lower surface roughness than its EBM counterparts. In-house measurements on SLM parts have revealed an average of absolute values for deviations from a central plane (*R*_*a*_) to be typically around 10 *μ*m while values ranging from 15 to 22 *μ*m have been reported for EBM [85]. These surfaces have good machinability and if required, parts can be finished to have surface roughness below 1.0 *μ*m. However, these inherent rough surfaces have proved to promote bone ingrowth in several investigations for both SLM and EBM [84, 86], while the surface chemistry has also been shown to conform to international standards [85]. Surfaces can also be made bioactive through postprocesses such as NaOH and HCl soaking to enhance its osteoconductive properties [87]. Utilisation of these as-built rough surfaces eliminates the need of extra postprocessing operations such as plasma spraying or grit blasting, reducing time, and waste in the process chain, resulting in more resource efficiency.

### 5.2. Tensile Properties

Not all literature specifies whether Ti-6Al-4V (grade 5) or Ti-6Al-4V ELI (grade 23), which has reduced content percentage of interstitial impurity atoms, has been used. In this review, only publications explicitly stating that grade 23 powder alloy was used are discussed. This alloy has been specifically selected for discussion on the basis of its FDA approval for production of cementless hip stems and its availability in powder form, conforming to international specifications, from the respective manufacturers of laser and electron beam melting machines. As-built titanium alloy parts generated with the SLM process have appropriate tensile and yield strengths but lack the necessary ductility. This is due to the resultant microstructure of the as-built material, which contains a metastable acicular martensitic (*α*′) structure [49]. With EBM, the as-built microstructure is dominated by an acicular *α* phase but also includes a small percentage of *β* phase, yielding a brittle part. Consequently, for both of these processes, ductility needs improvement with appropriate postprocessing operations. Tailoring of the microstructure by using different heat treatments can transform the SLM *α*′ phase to the more ductile *α* + *β* phase which render these materials comparable to their wrought counterparts [47, 52, 84, 88].

Table 3 summarizes some of these results and compares them to the tensile properties specified in ASTM F136-08 for wrought Ti-6V-4V ELI used in surgical implant manufacturing [46]. By applying suitable postprocesses, such as machining, polishing, and heat treatment strategies, the properties can be improved significantly. Depending on the intended application, trade-offs should be considered between the advantages of rough surfaces for bone ingrowth and required mechanical properties. Considering the above and the values in Table 3, it can be concluded that the tensile properties of Ti-6Al-4V ELI manufactured with SLM and EBM with appropriate postprocesses adhere to the standard specification as set out for the currently used wrought counterpart. An area of concern, however, regarding parts produced by these processes is fatigue strength.

### 5.3. Fatigue Strength

International standards for fatigue strength specify that the head region and neck region of a femoral stem have to withstand 10 million cycles of loading, as determined according to the test described in ISO 7206-6:1992 [89]. Characterisation of fatigue properties of SLM parts is, however, based on standardised specimen geometries and does not necessarily account for unusual geometries [83]. Properties from standardised specimens may vary to that of unconventional parts, due to the random distribution of porosity which is an inherent issue in the current state of the SLM manufacturing process [48, 83]. Techniques such as preheating of the powder bed may improve SLM processes [90]. Hot isostatic pressing (HIP) reduces porosity to almost negligible levels and is reported to increase fatigue strength [83]. Although promising results have been published [74], inconsistencies, likely due to random porosity due to suboptimal processing parameters [91], are preventing the successful commercialization of load-bearing devices.

## 6. Summary

### 6.1. Conclusions

Postoperative infections of THAs still remain a devastating complication. The high financial burden, morbidity, and psychological affliction further emphasize the need for continual improvement and evaluation of prevention and treatment alternatives. While promising results have been reported in recent literature, some issues still remain, allowing room for improvement, for example, the delivery of multiple doses through implants instead of employing once-off delivery strategies. With the evolution of AM technologies such as SLM and EBM which allow the direct manufacture of near net shape metallic parts, drug delivery functionality can be designed into cementless femoral stems to attend to aforementioned shortcomings. This however brings new challenges pertaining to the development of such implants, which fall under the classification of combination devices. For example, intraosseous delivered drugs may behave differently than that of intravenous or orally administered formulations. Bolus injections of 250 and 500 mg vancomycin, intraosseously, have been administered successfully without eliciting toxic side-effects [92] while, in a different study, no significant difference between intraosseous and intravenous administration of morphine sulphate was observed [93]. Drug delivery dosages can also be a combination of four formulations, diffusing at a defined rate while the reservoir is continuously replenished over six weeks by microsphere formulations, as described by Wang et al. [94]. Successful incorporation of such formulations will eliminate the need for administering multiple dosages, further enhancing the functionality of such implants. Such functional enhancements also bridge the once-off, single formulation release of current strategies to enable* in situ* administration of different drugs formulated according to release requirements.

### 6.2. Future Work

Various research avenues flow from the development of such a device. From a pharmaceutical perspective it is important that each new drug formulation be assessed to determine its optimal administration route and concentration, as this can differ depending on the biochemical, pharmacodynamic, and pharmacokinetic properties of each drug. The release mechanism as well as the internal implant design is directly affected by this.

Consequently, an iterative design process with simulation of mechanical properties should ensue to establish a feasible internal channel and reservoir design that strives to minimize the detrimental effect of material removal from the bulk structure. As such devices do not exist, there is a requirement for the development and evaluation of new process chains in order to demonstrate the most efficient manufacturing method for a given design. Such chains should be evaluated specifically for resource efficiency during small batch as well as its capability for series production.

It is evident that such an endeavour is inherently multidisciplinary in nature. Therefore, procedures for the integration of stakeholders across different disciplines into development teams which can provide a more comprehensive basis of knowledge inserts from various perspectives are important. A knowledge platform for effective collaboration would aid in elucidating interactions between aspects regarding involved disciplines which otherwise are often overlooked.

## Figures and Tables

**Table 1:** Pathogens isolated from prosthetic joint infections after total hip arthroplasty (THA) and total knee arthroplasty (TKA), adapted from [40].

Species or group	Percentage
*Staphylococcus aureus*	22
Polymicrobial composition	19
Coagulase-negative staphylococci (CNS)	19
Unidentified Gram-negative rods	20
*Streptococcus* spp.	9
Anaerobic bacteria	6
Microorganisms representing 5% and less of the cultured species:	
*Enterococcus *spp.	
*Corynebacterium *spp.	
*Listeria monocytogenes*	
*Mycobacterium tuberculosis*	
*Candida albicans*	
* Brucella suis*	
*Geotrichum *spp.	

**Table 2:** Commercially available, FDA-approved ALBC cements.

Product	Current distributor	Antibiotic	Concentration^a^	FDA approval
Simplex P	Stryker	Tobramycin	1.0 g	2003
Refobacin R^b^	Biomet	Gentamicin	0.5 g	2003
Palacos R+G^b^	Heraeus Medical	Gentamicin	0.5 g	2003
Smartset GHV	DePuy	Gentamicin	1.0 g	2004
VersaBond AB	Smith & Nephew	Gentamicin	1.0 g	2004
Cemex Genta	Exactech	Gentamicin	1.0 g	2004
CMW1	DePuy Orthopedics Inc.	Gentamicin	1.0 g	2005
Smartset GMV	DePuy Orthopedics Inc.	Gentamicin	1.0 g	2008

^a^Per 40 g bone cement.

^b^Originally developed as one product.

**Table 3:** SLM and EBM as-built (heat treated) tensile properties in comparison to ASTM F136-08.

Process/standard	Machine	Tensile strength [MPa]	Yield strength [MPa]	% elongation	Heat treatment	Reference
ASTM F136-08^a^	N/A	860	795	10 (minimum)	—	[46]
ASTM F136-08^b^	N/A	825	760	8 (minimum)	—	[46]
SLM	Concept laser M2	1211–1262 (950–1060)	1100–1150 (890–1030)	7.2–9 (6.5–11.7)	Recrystallisation annealing	[47]
SLM	Concept laser M2	±1200 (1000–1100)	>1000 (925–1000)	<10 (12–18)	HIP	[48]
SLM	LM-Q (custom built)	1267 ± 5 (948 ± 27)	1110 ± 9 (899 ± 27)	7.28 ± 1.12 (13.59 ± 0.32)	Beta annealing	[49]
EBM	Arcam A2	928	N/A	3%	—	[50]
EBM	Arcam S400	928 ± 9.8	869 ± 7.2	9.9 ± 1.7	—	[51]^c^
EBM	Arcam^d^	904 ± 6 (902 ± 8.7)	802 ± 7.9 (807 ± 8.4)	13.8 ± 0.9 (14.8 ± 0.5)	HIP	[52]

^a^Specified for diameters of 4.75 to under 44.45 mm.

^b^Specified for diameters of 44.45 to under 63.50 mm.

^c^Samples were machined for a smooth surface but no heat treatment was done.

^d^Machine not specified, and although not explicitly stated by the authors, it is suspected that as-built samples were first machined considering the elongation.
